# Supplementary material for: Nanoscopic distribution of VAChT and VGLUT3 in striatal cholinergic varicosities suggests colocalization and segregation of the two transporters in synaptic vesicles
Source: Front Mol Neurosci. 2022 Sep 13;15:991732. doi: 10.3389/fnmol.2022.991732 (PMC9513193; doi:10.3389/fnmol.2022.991732)
Supplement: Supplementary file 1 [file Data_Sheet_1.docx]

**Supplementary Data Sheet 1**

**Supplementary Method. Related to Figure 3. Method of calculation of the percentage of VAChT-alone, VGLUT3-alone or VAChT+VGLUT3 SVs in CINs varicosities.**

Estimation of the percentage of each subpopulation of SVs is based on results from Figure 2 combined with results from Figure 3.

1. First, in Figure 2**E**, VAChT and VGLUT3 were observed in purified SVs from the striatum of a WT mice. Quantification of these experiments showed a density of immunofluorescent spots of 0.603 ± 0.106 for VAChT and 0.441 ± 0.084 for VGLUT3 (in number of spots per µm^2^, Figure 2H). Double labeled spots (1-6 % of total CINs SVs) were neglected. This finding suggests that ≈ 60 % of CINs SVs are VAChT-positive whereas ≈ 40 % are VGLUT3-positive.

2. Second, from Figure 3A we calculated that 43 % of VAChT immunopositive spots (Figure 3E) have a NNDs <95 nm with VGLUT3 spots. This value likely corresponds mostly to transporters expressed by the same SVs.

3. Third, from experiments shown in Figure 3A, we estimated that 13% of VGLUT1-positive and 5% of VGLUT2-positive spots have a NND with a VGLUT3 spot <95nm; VGLUT1 and VGLUT3 or VGLUT2 and VGLUT3 being located in independent non cholinergic SVs (Figure 3G,I). From these values we estimate a value of “background” of (13 % + 5 %) / 2 = 9 %.

4. Once this “background” value has been subtracted, we obtain 34 % of VAChT and VGLUT3 immunopositive spots on the same SVs.

5. Therefore, it can be estimated that ≈34 % of CINs SVs co-express VGLUT3+VAChT whereas 66% express either VAChT or VGLUT3 alone.

6. If we apply the 60/40 ratio (determined in point 1) to this subpopulation of SVs, we obtain a distribution of ≈40 % of VAChT-alone and ≈26 % of VGLUT3-alone SVs.

In summary, based on these approximations it can be estimated that the proportion of VAChT+VGLUT3-positive SVs are ≈34 %, whereas VGLUT3 alone SVs are 26 % and VAChT alone SVs are 40 % (Figure 3J).
