# Supplementary material for: Nanoscopic distribution of VAChT and VGLUT3 in striatal cholinergic varicosities suggests colocalization and segregation of the two transporters in synaptic vesicles
Source: Front Mol Neurosci. 2022 Sep 13;15:991732. doi: 10.3389/fnmol.2022.991732 (PMC9513193; doi:10.3389/fnmol.2022.991732)
Supplement: Supplementary file 2 [file Table_1.pdf]

Supplementary Table 1 : Related to Figure 2. Quantification of the mean diameter of VGLUT3 and VAChT fluorescent spots with confocal or STED microscopy in the mouse dorsolateral striatum with confocal and STED microscopy.

**Mean diameter of fluorescent spots**<br>**Confocal vs STED**	
Wilcoxon matched-pairs signed rank test	
**VGLUT3**	**VAChT**
n=110 fluorescent spots	n=110 fluorescent spots
$p<0.0001$	$p<0.0001$
