# Supplementary material for: Nanoscopic distribution of VAChT and VGLUT3 in striatal cholinergic varicosities suggests colocalization and segregation of the two transporters in synaptic vesicles
Source: Front Mol Neurosci. 2022 Sep 13;15:991732. doi: 10.3389/fnmol.2022.991732 (PMC9513193; doi:10.3389/fnmol.2022.991732)
Supplement: Supplementary file 3 [file Table_2.pdf]

Supplementary Table 2 : Related to Supplementary Figure 2. Quantification of the mean diameter of fluorescent spots with STED microscopy before and after deconvolution and Gauss blur filter.

**Mean diameter of fluorescent spots – STED<br>Before and after deconvolution**	
Wilcoxon matched-pairs signed rank test	
**VAChT**	**VGLUT3**
n=109 fluorescent spots	n=100 fluorescent spots
p<0.0001	p<0.0001
