# Supplementary material for: Nanoscopic distribution of VAChT and VGLUT3 in striatal cholinergic varicosities suggests colocalization and segregation of the two transporters in synaptic vesicles
Source: Front Mol Neurosci. 2022 Sep 13;15:991732. doi: 10.3389/fnmol.2022.991732 (PMC9513193; doi:10.3389/fnmol.2022.991732)
Supplement: Supplementary file 4 [file Table_3.pdf]

Supplementary Table 3 : Related to Figure 2. Quantification of the mean number of VGLUT3, VAChT and VAChT + VGLUT3 fluorescent spots per surface of microscope slide with STED microscopy in isolated striatal synaptic vesicles from VGLUT3$^{+/+}$ and VGLUT3$^{-/-}$ mice.

Kruskal Wallis test, Dunn's post hoc test					
**VAChT fluorescent spots**					
n=19 microscopic fields ($625 \mu m^2$)					
VGLUT3+/+	VGLUT3-/-	VGLUT3+/+	No anti-VGLUT3 antibody	VGLUT3-/-	No anti-VGLUT3 antibody
nb spots = 6348	nb spots = 3927	nb spots = 6348	nb spots = 5233	nb spots = 3927	nb spots = 5233
$P<0.0001$		$P<0.0001$		p=0,6378	
**VGLUT3 fluorescent spots**					
n=19 microscopic fields ($625 \mu m^2$)					
VGLUT3+/+	VGLUT3-/-	nb spots =4301	No anti-VGLUT3 antibody	VGLUT3-/-	No anti-VGLUT3 antibody
nb spots =4301	nb spots = 293	nb spots =4301	nb spots = 381	nb spots = 293	nb spots = 381
$p<0.0001$		$p<0.0001$		$p>0.9999$	
**VAChT + VGLUT3 fluorescent spots**					
n=19 microscopic fields ($625 \mu m^2$)					
VGLUT3$^{+/+}$	VGLUT3$^{–/–}$	VGLUT3$^{+/+}$	No anti-VGLUT3 antibody	VGLUT3$^{–/–}$	No anti-VGLUT3 antibody
nb spots = 696	nb spots = 40	nb spots = 696	nb spots = 28	nb spots = 40	nb spots = 28
$p<0.0001$		$p<0.0001$		$p>0.9999$	
