# Supplementary material for: Nanoscopic distribution of VAChT and VGLUT3 in striatal cholinergic varicosities suggests colocalization and segregation of the two transporters in synaptic vesicles
Source: Front Mol Neurosci. 2022 Sep 13;15:991732. doi: 10.3389/fnmol.2022.991732 (PMC9513193; doi:10.3389/fnmol.2022.991732)
Supplement: Supplementary file 5 [file Table_4.pdf]

Supplementary Table 4 : Related to Figure 3. Frequency distributions of the NND between VAChT and VGLUT3, VGLUT1 and VGLUT3, VGLUT1 and VGLUT3-immunofluorescent spots. Observation under a STED microscope of isolated striatal synaptic vesicles.

**Frequency distributions of the NND**		
Kolmogorov-Smirnov test		
n=7048 (VAChT and VGLUT3) and 6017 (VGLUT1 and VGLUT3) pairs of spots	n=7048 (VAChT and VGLUT3) and 6827 (VGLUT2 and VGLUT3) pairs of spots	n=6017 (VGLUT1 and VGLUT3) pairs of spots and 6827 (VGLUT2 and VGLUT3) pairs of spots
**VAChT and VGLUT3 vs. VGLUT1 and VGLUT3**	**VAChT and VGLUT3 vs. VGLUT2 and VGLUT3**	**VGLUT1 and VGLUT3 vs. VGLUT2 and VGLUT3**
$p<0.0001$	$p<0.0001$	$p<0.0001$
