# Supplementary material for: Nanoscopic distribution of VAChT and VGLUT3 in striatal cholinergic varicosities suggests colocalization and segregation of the two transporters in synaptic vesicles
Source: Front Mol Neurosci. 2022 Sep 13;15:991732. doi: 10.3389/fnmol.2022.991732 (PMC9513193; doi:10.3389/fnmol.2022.991732)
Supplement: Supplementary file 6 [file Image_1.pdf]

**Supplementary Figure 1 : Related to Figure 2. Effect of deconvolution on VAChT- and VGLUT3-immunofluorescent spot resolution. (A)** Representative raw STED image of VAChT and VGLUT3-immunofluorescent spots. **(B)** The same raw image after post-processing using the Gaussian blur filter in ImageJ with a radius of 1 pixel ($\sigma$=1). **(C,D)** Images after post-processing using Huygens Deconvolution with or without Gaussian blur filter. **(E,F)** Quantification of the mean diameter of VAChT- and VGLUT3- fluorescent spots with STED microscopy before and after deconvolution and Gauss blur filter. Quantification of 109 VAChT- and 100 VGLUT3- fluorescent spots. Wilcoxon matched-pairs signed rank test, **$p$<0.0001. Fluorochromes: Alexa 594 (VAChT), Abberior Star 635P (VGLUT3).

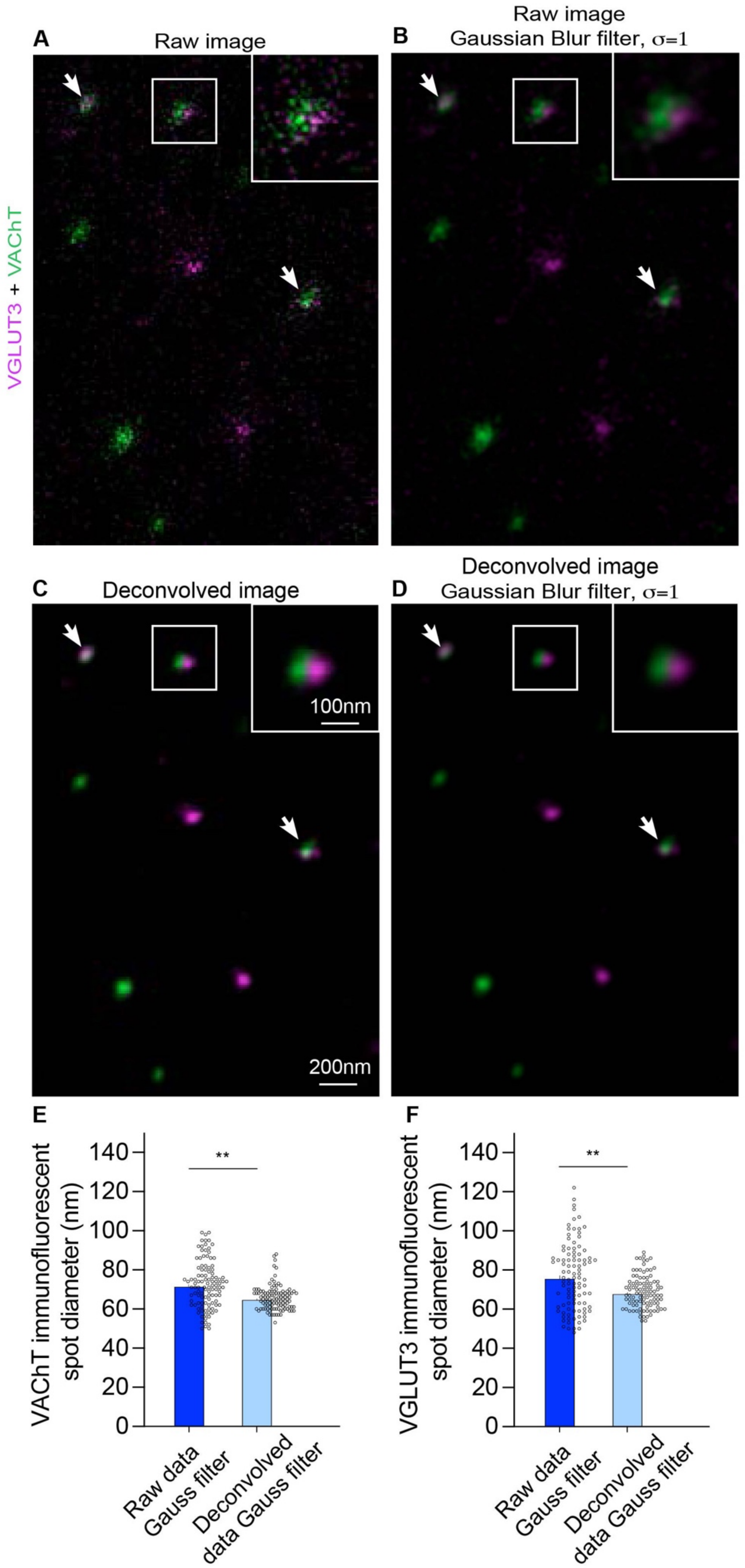

A
Raw image
B
Raw image
Gaussian Blur filter, σ=1
VGLUT3 + VAChT
C
Deconvolved image
100nm
200nm
D
Deconvolved image
Gaussian Blur filter, σ=1
E
VAChT immunofluorescent spot diameter (nm)
140
120
100
80
60
40
20
0
**
Raw data Gauss filter
Deconvolved data Gauss filter
F
VGLUT3 immunofluorescent spot diameter (nm)
140
120
100
80
60
40
20
0
**
Raw data Gauss filter
Deconvolved data Gauss filter
