# Supplementary material for: Nanoscopic distribution of VAChT and VGLUT3 in striatal cholinergic varicosities suggests colocalization and segregation of the two transporters in synaptic vesicles
Source: Front Mol Neurosci. 2022 Sep 13;15:991732. doi: 10.3389/fnmol.2022.991732 (PMC9513193; doi:10.3389/fnmol.2022.991732)

Supplementary Figure 2: Related to Figure 2. Immunohistochemical detection of VAChT on isolated mouse striatal vesicles with confocal and STED microscopy. (A,A') Immunofluorescent detection of VAChT on isolated SVs from mouse striatum with confocal **(A)** or STED **(A')** microscopy. **(B)** Quantification of the mean diameter of fluorescent spots with confocal or STED microscopy. Quantification of 110 fluorescent spots. Wilcoxon matched-pairs signed rank test, **p<0.0001. Fluorochrome: Alexa 594.

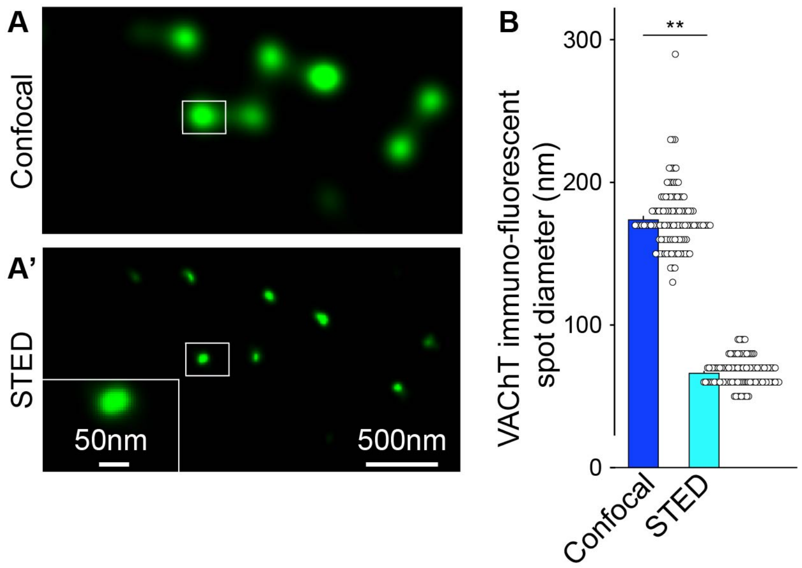

Supplement: Supplementary file 7 [file Image_2.pdf]
