# Supplementary material for: Nanoscopic distribution of VAChT and VGLUT3 in striatal cholinergic varicosities suggests colocalization and segregation of the two transporters in synaptic vesicles
Source: Front Mol Neurosci. 2022 Sep 13;15:991732. doi: 10.3389/fnmol.2022.991732 (PMC9513193; doi:10.3389/fnmol.2022.991732)

Supplementary Figure 3 : Related to Figure 3. Models to evaluate the distribution of SVs in preparations of isolated striatal SVs and based-NND quantification. (A) Clusterization model. Schematic representation of VAChT- and VGLUT3-immunopositive spots in a clustered distribution. The circle represents the area of analysis and is similar to the surface of a CINs varicosity (500 nm in diameter). **(B)** Dispersion model. Schematic representation of VAChT- and VGLUT3-immunopositive spots in a dispersed distribution. **(C)** Quantification of the proportion of the first, second, third or fourth NNDs below or above 500 nm using the DiAna plugin. Quantification from 1506 pairs of VAChT-VGLUT3 spots, from 3 experiments.

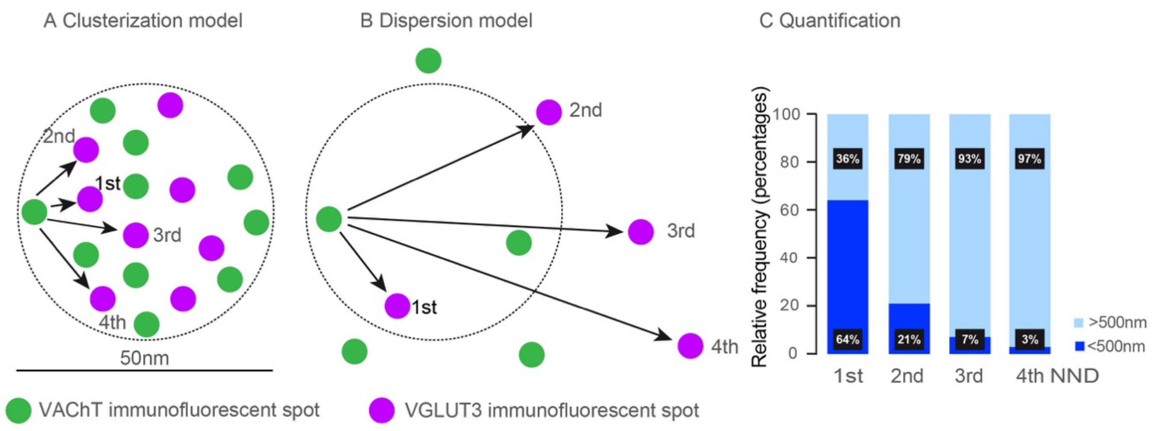

Supplement: Supplementary file 8 [file Image_3.pdf]
